# The rational design of iron-sulfur cluster binding site for prolonged stability in magnetoreceptor MagR

**DOI:** 10.3389/fmolb.2022.1051943

**Published:** 2022-11-10

**Authors:** Tianyang Tong, Yajie Zhou, Fan Fei, Xiujuan Zhou, Zhen Guo, Shun Wang, Jing Zhang, Peng Zhang, Tiantian Cai, Guohui Li, Yuebin Zhang, Junfeng Wang, Can Xie

**Affiliations:** ^1^ Department of Anatomy, School of Basic Medicine, Anhui Medical University, Hefei, Anhui, China; ^2^ High Magnetic Field Laboratory, Hefei Institutes of Physical Science, Chinese Academy of Sciences, Science Island, Hefei, China; ^3^ Institutes of Physical Science and Information Technology, Anhui University, Hefei, Anhui, China; ^4^ Science Island Branch of Graduate School, University of Science and Technology of China, Hefei, Anhui, China; ^5^ School of Life Sciences, Peking University, Beijing, China; ^6^ Department of Biological Chemistry and Molecular Pharmacology, Harvard Medical School, Boston, MA, United States; ^7^ State Key Laboratory of Molecular Reaction Dynamics, Dalian Institute of Chemical Physics, Chinese Academy of Sciences, Dalian, China; ^8^ International Magnetobiology Frontier Research Center, Science Island, Hefei, China

**Keywords:** iron-sulfur cluster, magnetoreceptor, MagR, rational design, thermostability

## Abstract

Iron-sulfur proteins play essential roles in a wide variety of cellular processes such as respiration, photosynthesis, nitrogen fixation and magnetoreception. The stability of iron-sulfur clusters varies significantly between anaerobic and aerobic conditions due to their intrinsic sensitivity to oxygen. Iron-sulfur proteins are well suited to various practical applications as molecular redox sensors or molecular “wires” for electron transfer. Various technologies have been developed recently using one particular iron-sulfur protein, MagR, as a magnetic tag. However, the limited protein stability and low magnetic sensitivity of MagR hindered its wide application. Here in this study, the iron-sulfur binding site of pigeon clMagR was rationally re-designed. One such mutation, T57C in pigeon MagR, showed improved iron-sulfur binding efficiency and higher iron content, as well as prolonged thermostability. Thus, clMagR^T57C^ can serve as a prototype for further design of more stable and sensitive magnetic toolbox for magnetogenetics in the future.

## Introduction

Iron-sulfur clusters are essential cofactors consisting of ferrous (Fe^2+^) or ferric (Fe^3+^) iron and sulfide (S^2-^) ions and comprise the largest class of metalloproteins present in almost all organisms. The most common types of iron-sulfur clusters are rhombic [2Fe-2S], cubic [3Fe-4S] and cubic [4Fe-4S] (Kiley and Beinert, 2003; Hinton et al., 2022). Iron-sulfur clusters are usually bind to cysteine (Cys) residues through iron ions. In addition, histidine (His), aspartic acid (Asp) and glutamic acid (Glu) residues can also serve as coordination bonds to iron-sulfur clusters and exhibit unique functions (Wiley et al., 2007; Zeng et al., 2008; Gruner et al., 2011; Volbeda et al., 2019).

Electron transfer is perhaps the most obvious function which has been identified since early 1960s in photosynthesis and respiration systems (Beinert, 1960; Mortenson et al., 1962). Later, a wide variety of biological functions have emerged for these clusters, including nitrogen fixation, DNA replication and repair (Kiley and Beinert, 2003; Johnson et al., 2005; Fontecave, 2006; Mettert and Kiley, 2015; Rouault, 2015). In 2015, Qin et al. reported an iron-sulfur protein MagR (Magnetoreceptor, originally named IscA) played essential roles in animal magnetoreception through the interaction with cryptochrome (Cry) (Qin et al., 2016). The MagR/Cry-based biocompass model (Qin et al., 2016; Xie, 2022) combined the concept of both the magnetite-based mechanism (Hsu et al., 2007; Eder et al., 2012; Wiltschko and Wiltschko, 2013; Monteil and Lefevre, 2020; Schuler et al., 2020) and radical-pair based mechanism (Ritz et al., 2000; Liedvogel et al., 2007; Gegear et al., 2008; Lau et al., 2012; Wiltschko and Wiltschko, 2014; Xu et al., 2021), thus provided an solution for both polarity detection and inclination detection. The iron-sulfur cluster of MagR is required for the assembly of the MagR/Cry protein complex (Qin et al., 2016), and has been suggested to mediate the long range intermolecular electron transport chain in MagR/Cry complex (Qin et al., 2016; Xie, 2022), and contribute to the intrinsic magnetic moment of MagR and MagR/Cry complex (Guo et al., 2021). Furthermore, two different types of iron-sulfur clusters, [2Fe-2S] and [3Fe-4S], have been identified in MagR and may serve as a magnetic switch to modulate the magnetic property of MagR (Guo et al., 2021).

In pace with the growing interest in elucidating the underlying mechanism of MagR as a putative magnetoreceptor, various technologies have been developed recently using MagR as a magnetic tag (Jiang et al., 2017; Xue et al., 2020; Kang et al., 2021). Biological manipulation *via* magnetic fields, which is also refer to as magnetogenetics, has been a pre-eminent goal for scientists. It is especially appealing for *in vivo* applications since magnetic field can penetrate deep into tissues, which allow non-invasive remote modulation of biological processes possible. This is achieved by fusing a magnetic tag to a mechanically sensitive ion channel such as TRPV4, and then applying magnetic field to exert magnetic force on the channel to open up the associated channel and activate biological systems, such as neuronal functions. To engineer MagR as a suitable actuator for magnetogenetics, a rationally re-designed MagR with improved stability and higher magnetic sensitivity is required to overcome the thermal fluctuations at room temperature. Therefore, there has been a major effort to re-design a better MagR for applications, for example, a single-chain tetramer MagR was designed as a building block to increase the protein self-assembly efficiency and thus increase the magnetic sensitivity by polymerization (Yang et al., 2022). Another particular approach is to design a more stable binding site to host the iron-sulfur cluster since iron-sulfur clusters are critical for the magnetism of MagR, as described in this paper.

The stability of iron-sulfur clusters varies significantly between anaerobic and aerobic conditions due to their intrinsic sensitivity to oxygen. When they are harbored in proteins, the stability is primarily dependent on the microenvironments within a biomolecular structure, such as the oxygen accessibility to the clusters, and coordination bonds of the clusters, et al. (Meyer, 2008). Iron-sulfur clusters in some proteins (e.g., thermophilic Fd, a thermostable [2Fe-2S] ferredoxin from hyperthermophilic bacterium *Aquifex aeolicus*) are unusually stable for weeks even exposed in air (Mitou et al., 2003), while in many or in the majority of iron-sulfur proteins are very sensitive to oxygen and only stable for tens of seconds (e.g., nitrogenase) (Eady et al., 1972). As for pigeon (*Columba livia*) MagR (clMagR), the iron-sulfur cluster is normally stable for 4–5 days at room temperature (298 K) and 7 days at 4°C (277 K).

To further stabilize the iron-sulfur cluster binding in MagR, here in this study, the binding site was rationally re-designed based on the 3D structural model of MagR. Two hotspot regions located close to the iron-sulfur binding site and around E128 and T57/R58 respectively were identified. Site-directed mutagenesis were then designed aiming to stabilize the iron-sulfur cluster binding. One such mutation, T57C in clMagR, has been identified with increased iron-sulfur cluster binding half-life. The prolonged thermostability of clMagR^T57C^ makes it suitable to serve as a prototype for further fine-tuning as a magnetic actuator for controlling biological processes in the future.

## Results

### E128 is not a potential ligand of iron-sulfur cluster in pigeon MagR

**FIGURE 1:**
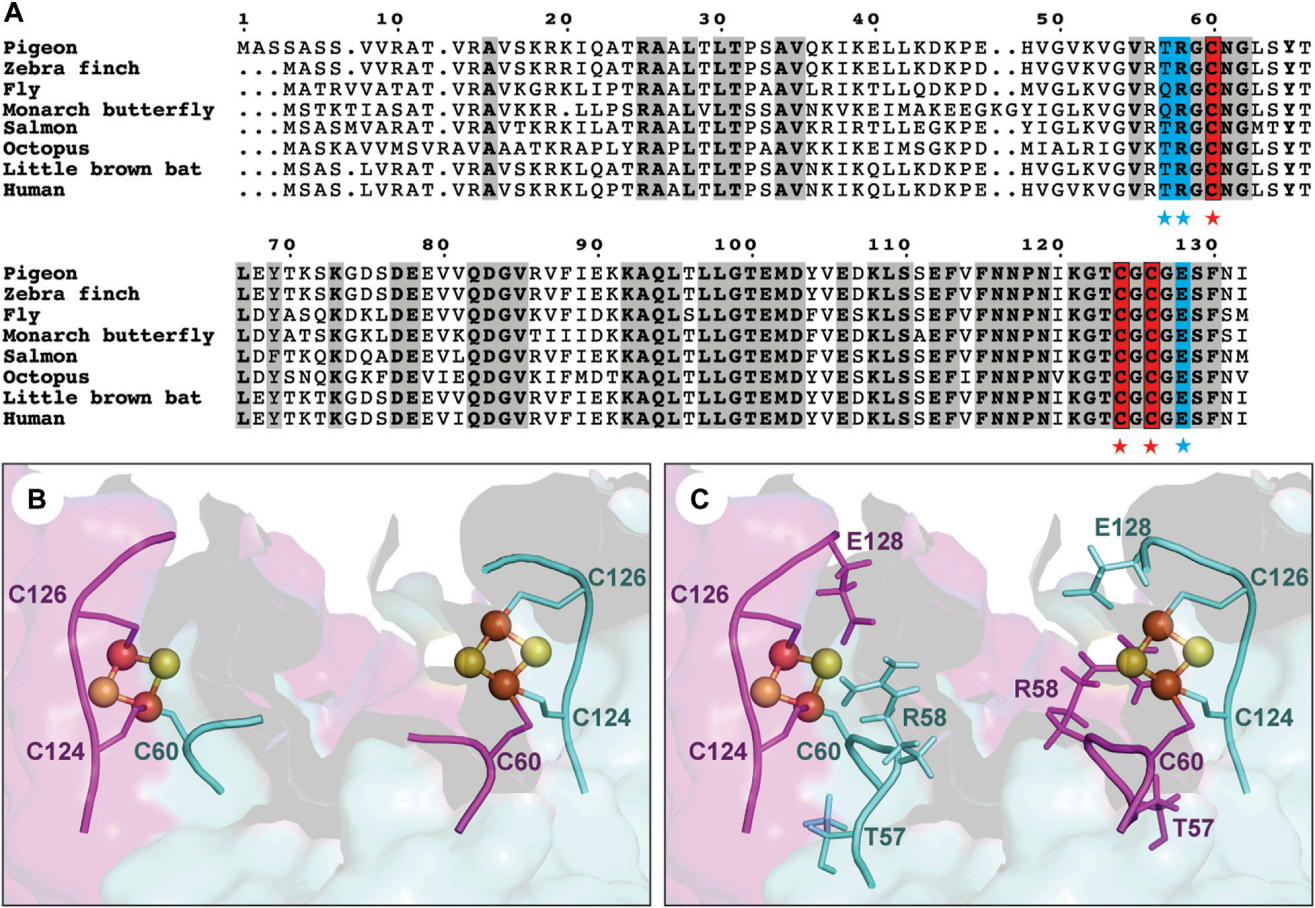
Sequence conservation and structural feature of the iron-sulfur cluster binding site of clMagR. **(A)** Sequence alignment of MagR from eight representative species. Three conserved cysteines (C60, C124 and C126) forming the iron-sulfur cluster binding site are highlighted in red background and marked by red stars. Residues are mutated in this study are shown in blue background and marked by blue stars. The conserved residues are shown in bold and grey background. **(B)** Six conserved cysteines from clMagR dimer form two iron-sulfur cluster binding sites in structural model. **(C)** Three nearby residues (T57, R58 and E128) located in two hotspot regions could potentially coordinate with iron-sulfur binding sites are shown in structural model.

Three highly conserved cysteines (Cys60, Cys124, Cys126) have been identified to bind two types of iron-sulfur clusters, [2Fe-2S] and [3Fe-4S], in freshly purified pigeon MagR in aerobic conditions (Guo et al., 2021) (Figures 1A,B). A three-dimensional structural model of pigeon MagR was initially generated based on bacterial homologous IscA structure (PDB ID:1R94) as described previously (Qin et al., 2016), then fine-tuned using the MODELLER homology modeling package (Yang et al., 2022). A careful analysis of the structural model revealed a hotspot residue, glutamic acid (E128), located close (around 3.6 Å) to the iron-sulfur cluster (Figure 1C and Supplementary Figure S1), leading to the question if E128A serve as a potential ligand of iron-sulfur cluster? May or may not, would it be possible to design a more stable binding site by mutating E128 to cysteine residue for iron-sulfur cluster coordination?

**FIGURE 2:**
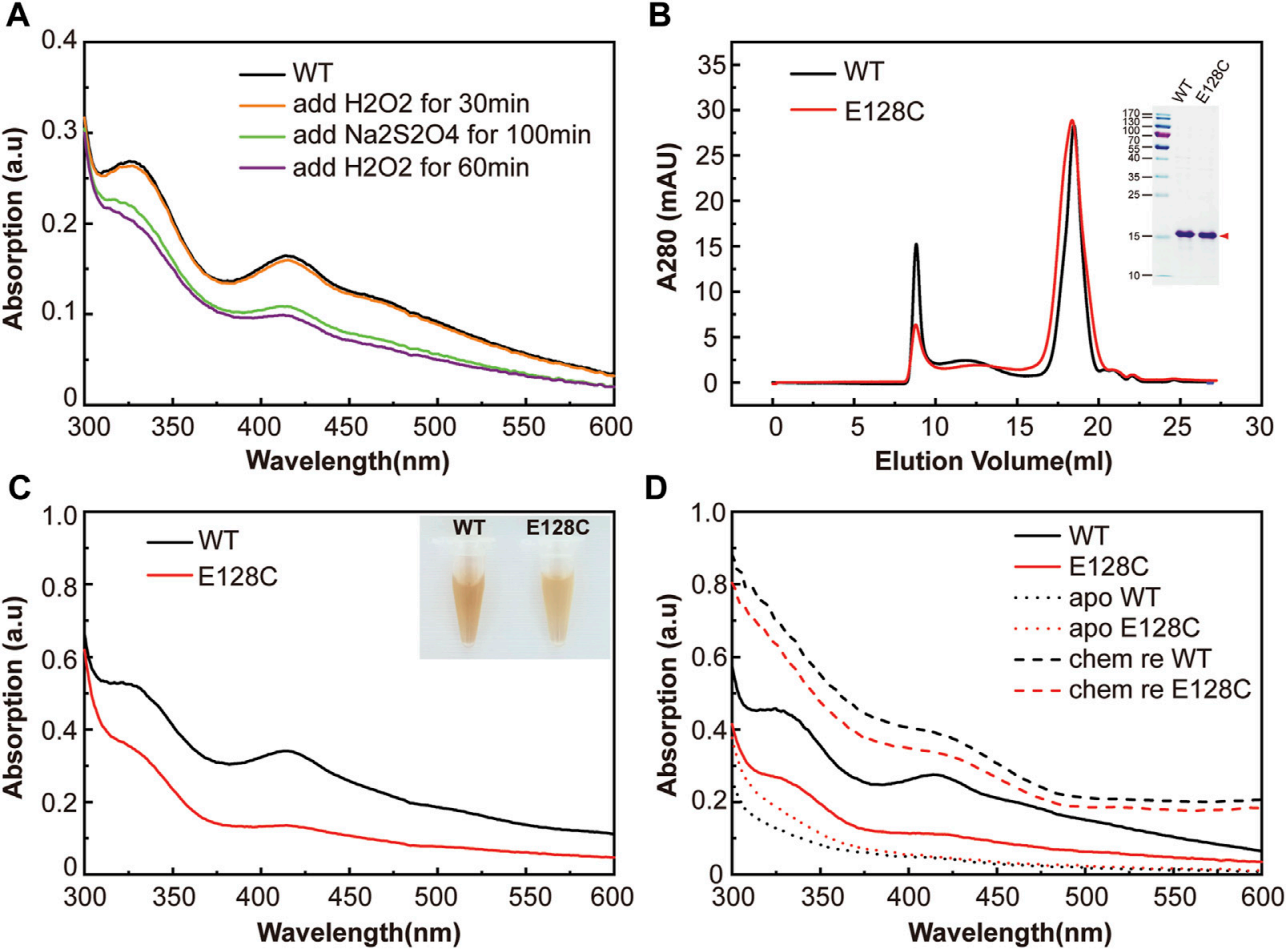
The functional role of E128 in clMagR iron-sulfur cluster binding. **(A)** The redox cycling experiment of clMagR^WT^ protein monitored by UV-Vis absorption spectrum. As isolated protein (WT) was shown as black line, subsequently H_2_O_2_ oxidized protein, Na_2_S_2_O_4_ reduced protein and H_2_O_2_ re-oxidized protein were shown as orange, green and purple lines, respectively. **(B)** Size-exclusion chromatography of purified wild type clMagR protein (WT, black line) and its mutant clMagR^E128C^ (abbreviated as E128C, red line). SDS-PAGEs of protein preparation are shown as inserts. **(C)** UV–Vis absorption spectrum of purified clMagR (WT) and its mutant (E128C), with the same color scheme as in **(A) (D)** UV-Vis absorption spectrum of chemically reconstituted clMagR (WT) and its mutant (E128C). As-isolated proteins were shown as solid lines, apo proteins with iron-sulfur cluster removal were shown as dotted lines, and chemically reconstituted proteins (labeled as chem re) were shown as dashed lines.

Not only cysteine, but also histidine and glutamate acid residues could potentially coordinate [2Fe−2S] cluster binding. As previously reported in the transcription regulator RsrR, glutamic acid and histidine both served as ligands of iron-sulfur cluster and played critical roles in sensing the redox status of the cell *via* the facile cycling of the [2Fe−2S] cluster between +2 and +1 states (Volbeda et al., 2019). To further investigate if E128 in pigeon MagR could potentially function similarly, we performed redox cycling of clMagR protein. Briefly, we firstly measured the Ultraviolet–visible (UV-Vis) absorption spectra of as purified clMagR^WT^ (Figure 2A, black line), followed the subsequent addition of stoichiometric hydrogen peroxide (H_2_O_2_) and incubated at 4°C for 30 min (orange line), then, adding stoichiometric sodium dithionite and incubated at 4°C for about 100 min (green line). Finally, the samples were re-added with stoichiometric hydrogen peroxide (H_2_O_2_) and incubated at 4°C for 60 min (purple line). The results showed that re-adding H_2_O_2_ after clMagR^WT^ reduction did not re-oxidize the protein, indicating that clMagR is lacking the glutamate acid-mediated [2Fe−2S] redox cycle.

We then tested if substituting E128 to cysteine residue could enhance or stabilize the iron-sulfur cluster. A single amino acid substitution in clMagR (clMagR^E128C^) was generated to test this hypothesis, and clMagR^E128A^ was used as a control to compare the effect on iron-sulfur binding by mutagenesis. Both mutants and wild-type proteins were expressed and purified to homogeneity. Size-exclusion chromatography and SDS-PAGE showed similar conformations and molecular weight among clMagR^E128A^, clMagR^E128C^ and clMagR^WT^ (Figure 2B and Supplementary Figure S2A), indicating the mutants preserved the correct protein folding of MagR. The purified clMagR^E128C^ and clMagR^E128A^ proteins showed similar characteristic brownish color compared with clMagR^WT^ in solution (Figure 2C and Supplementary Figure S2B). Ultraviolet–visible (UV–Vis) spectrum from 300 nm to 600 nm wavelength showing absorption peaks at 320 and 420 nm, and a shoulder at 460 nm (Dailey et al., 1994; Netz et al., 2016; Guo et al., 2021), further confirmed that the iron-sulfur cluster incorporation was not affected by mutation (Figure 2C and Supplementary Figure S2B). However, lighter coloration and reduced UV-Vis absorption peaks (320 nm, 420 nm, 460 nm) of clMagR^E128C^ protein were observed, suggesting the impaired iron-sulfur cluster binding by mutation. One possibility could be the additional cysteine (-SH) in clMagR^E128C^ may interfere with the assembly of iron-sulfur clusters or lead to local structural instability in iron-sulfur binding site.

Wild type clMagR and its E128 mutant protein were chemically reconstituted respectively to check the iron-sulfur binding capacity *in vitro* (Figure 2D and Supplementary Figure S2C)*.* Briefly, the iron-sulfur clusters of MagR were removed by incubating with sodium dithionite and EDTA, and confirmed by UV-Vis spectrum (apo clMagR, dotted line). Then, fresh ferrous ammonium sulfate (Fe(NH_4_)_2_(SO_4_)_2_) and sodium sulfide (Na_2_S) were added to the apo protein to reconstitute iron-sulfur clusters. The iron-sulfur cluster constitution was verified by UV-Vis spectrum (chem re clMagR, dashed line). The UV absorption of reconstituted proteins appeared to be significantly higher than that of as-isolated clMagR. The successful reconstitution of clMagR and its E128 mutants (especially clMagR^E128A^) suggested that E128 might not a ligand of iron-sulfur cluster and should not be involved in iron-sulfur binding directly.

Highly conserved cysteine rich motifs such as CXC motif (e.g., C124 and C126 in pigeon MagR) and CX_2_C motif were often found in iron-sulfur binding site. We designed clMagR^G125_C126insK^ mutation to covert CXC motif to CX_2_C motif around this region (Supplementary Figure S1), but also found the iron-sulfur binding efficiency decreased as well (Supplementary Figure S3).

Taken together, we concluded that E128 was not a potential ligand of iron-sulfur cluster in clMagR, and mutating E128 to cysteine residue neither increased the binding efficiency nor affected the type of iron-sulfur clusters in clMagR.

### T57C mutation improved the iron-sulfur cluster binding in pigeon MagR

Based on the structural model of clMagR, the second hotspot region was found located around the conserved C60 residue (Figure 1C). We analyzed the known iron-sulfur cluster binding protein structures from RCSB Protein Data Bank and performed sequence alignment for the CX_2_C motif of iron-sulfur binding sites based on known structures (PDB ID: 3ZXS, 4Z3Y, 5C4I, 4UNF, 4S23, 1DUR). We mainly focused on CXGC motif, since C60 has been identified as the ligand of iron-sulfur cluster and has a nearby glycine residue in position 59 in clMagR (Supplementary Figure S1) (Qin et al., 2016; Guo et al., 2021). Two mutants, clMagR^T57C^ and clMagR^T57_R58insC^, were then designed aiming to reconstruction of an artificial CXGC motif in clMagR and to provide additional ligand for [2Fe−2S] binding. clMagR^R58C^ was also generated as a control of clMagR^T57C^.

**FIGURE 3:**
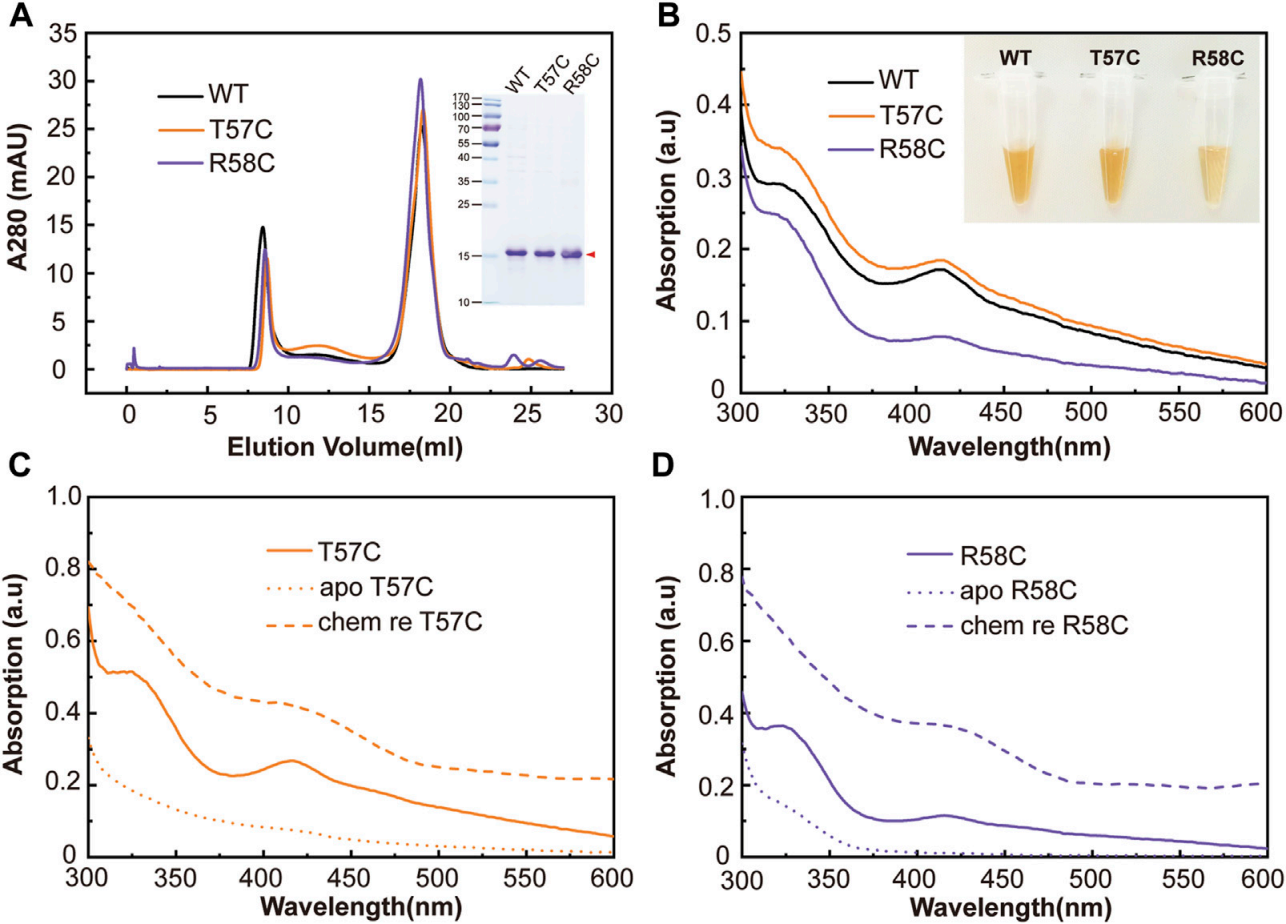
Mutations designed around C60 region in clMagR identified T57C increased iron-sulfur cluster binding. **(A)** Size-exclusion chromatography of purified wild type clMagR (WT, black line) and its mutants, clMagR^T57C^ (abbreviated as T57C, orange line) and clMagR^R58C^ (abbreviated as R58C, purple line). SDS-PAGEs of protein preparation are shown as inserts. **(B)** UV–Vis absorption spectrum of purified clMagR (WT) and its mutants (T57C and R58C), with the same color scheme as in **(A) (C,D)** UV-Vis absorption spectrum of chemically reconstituted clMagR mutants, T57C **(C)** and R58C **(D)**. The data for chemically reconstituted wild type clMagR protein was shown in Figure 2D. As-isolated proteins were shown as solid lines, apo proteins with iron-sulfur cluster removal were shown as dotted lines, and chemically reconstituted proteins (labeled as chem re) were shown as dashed lines.

All three mutants were expressed and purified as described above. Size-exclusion chromatography and SDS-PAGE showed similar conformations and molecular weights compared with wild type clMagR (Figure 3A). Freshly purified clMagR^WT^ protein and three mutants (clMagR^T57C^, clMagR^R58C^ and clMagR^T57_R58insC^) all showed brown color, indicating the iron-sulfur cluster binding in purified proteins. Purified clMagR^T57C^ showed slightly thicker color, whereas the color of clMagR^R58C^ and clMagR^T57_R58insC^ protein were relatively lighter compared with that of clMagR^WT^ at the same concentration (Figure 3B and Supplementary Figure S3). Consistently, the UV-Vis absorption peaks (320 nm, 420 nm, 460 nm) of clMagR^T57C^ were higher than those of clMagR^WT^, but clMagR^R58C^ showed significantly decreased UV-Vis absorption (Figure 3B), and clMagR^T57_R58insC^ even showed very different UV-Vis spectrum indicating the iron-sulfur cluster binding site was almost abolished (Supplementary Figure S3). After chemical reconstitution to incorporate iron-sulfur clusters, both the chemically reconstituted clMagR^T57C^ (Figure 3C, dashed line) and clMagR^WT^ (Figure 2D, dashed line) showed similar UV-Vis absorption, which is higher than that of clMagR^R58C^ (Figure 3D, dashed line), indicating the iron-sulfur binding probably was impaired by R58C substitution.

**FIGURE 4:**
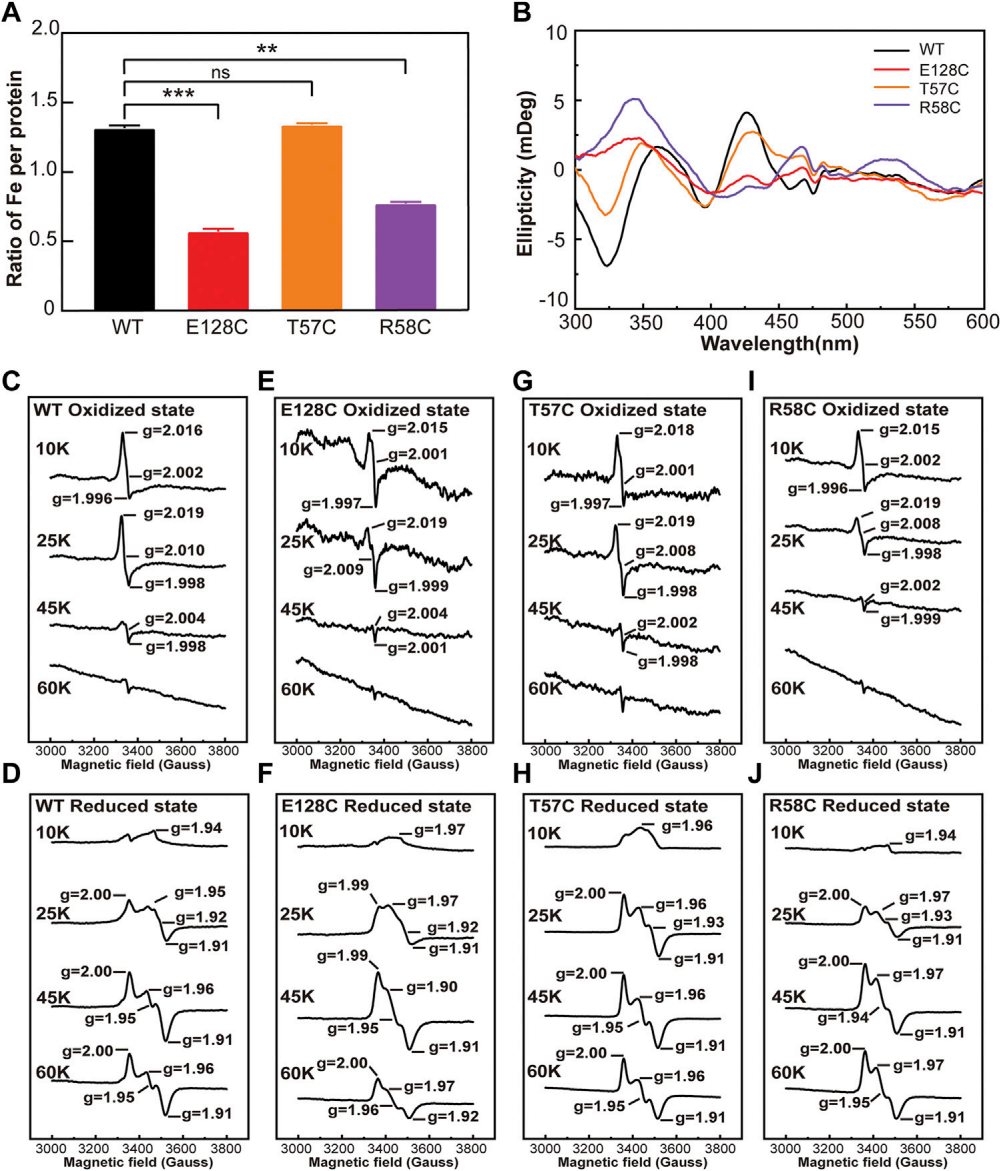
The iron content and the type of iron-sulfur cluster of wild type clMagR and its mutants. **(A)** Iron content of purified wild type clMagR (WT, black) and its mutants measured by Ferrozine assay and presented as the ratio of iron atoms in each protein. Student’s t-test: **, *p* < 0.01; ***, *p* < 0.001; ns, *p* >0.05, “ns” indicate no significant differences. Error bars: SD of three experiments. **(B)** Circular dichroism (CD) spectrum of wild type clMagR and its mutants. **(C–J)** EPR spectrum of wild type clMagR (WT, **C,D**) and its mutants, E128C **(E,F)**, T57C **(G,H)** and R58C **(I,J)**. EPR were measured both at oxidized (upper) status and reduced status (lower), and recorded at different temperatures (10 K, 25 K, 45 K and 60 K). Asterisks above bars indicate statistically significant differences between treatments at.

The total iron content of clMagR^WT^ and its mutants was measured by Ferrozine assay, an accurate and rapid method of the quantitation of iron in biological systems (Im et al., 2013; Landry et al., 2013; de Mello Gabriel et al., 2021). It is obvious that all mutations except T57C decreased the iron content in clMagR, and T57C even showed increased iron content compared that of wild type clMagR (Figure 4A), which was consistent with the UV-Vis spectrum results and the coloration of the purified proteins as well.

Circular dichroism (CD) spectroscopy was further applied to characterize the types of iron–sulfur cluster and their protein environments. As shown in Figure 4B and Supplementary Figure S2D, both wild type clMagR and its mutants showed distinctly positive peaks at 371 nm and 426 nm and three negative peaks at 324 nm, 396 nm, and 463 nm, respectively, suggesting the presence of [2Fe–2S] cluster (Azam et al., 2020). Since [4Fe–4S] or [3Fe–4S] clusters usually exhibit negligible CD intensity compared to [2Fe–2S], thus CD spectroscopy cannot exclude the possible existence of [4Fe–4S] or [3Fe–4S].

Electron paramagnetic resonance (EPR) spectroscopy was then used to further identify the iron-sulfur cluster types in different states of wild type clMagR protein and its mutants (Figures 4C–J and Supplementary Figure S4). The oxidized clMagR^WT^ protein was characterized by a rhombic EPR signal with g values at g_1_ = 2.016, g_2_ = 2.002, and g_3_ = 1.996, and disappeared at 45 K (Figure 4C), suggesting the presence of [3Fe–4S] (Rothery et al., 2001; Hoppe et al., 2011; Pandelia et al., 2011; Liu et al., 2013; Guo et al., 2021). Moreover, after reduction with sodium dithionite, the EPR signal from the [2Fe-2S] cluster can be observed at 45K and 60K (Figure 4D) (Netz et al., 2016; Zhang et al., 2017; Guo et al., 2021), which is consistent with previous report (Guo et al., 2021) . Thus, two distinct iron–sulfur clusters, [2Fe–2S] and [3Fe–4S], were assigned by EPR spectroscopy of clMagR^WT^. As for the mutants, the g values of clMagR^E128A^, clMagR^E128C^, clMagR^T57C^ and clMagR^R58C^ were all similar with clMagR^WT^ both in oxidized and reduced states (Figures 4E–J and Supplementary Figure S4), indicating that mutation in these positions did not affect the type of iron-sulfur cluster clMagR bound. The EPR spectral signal of clMagR^T57C^ was stronger than that of clMagR^WT^ at 25 K in the reduced state (Figure 4H), indicating that the iron-sulfur clusters binding in clMagR^T57C^ might be improved at this temperature. Whether clMagR^T57C^ has enhanced stability at ambient temperature remains unknown and further investigation is required.

Taking together, the stronger EPR signal, the thicker coloration of purified protein, the increased UV-Vis absorption and Ferrozine staining in clMagR^T57C^ suggested that a more stabilized iron-sulfur cluster binding, thus clMagR^T57C^ might serve as a valuable candidate for further exploration.

### Prolonged iron-sulfur cluster stability in clMagR^T57C^

**FIGURE 5:**
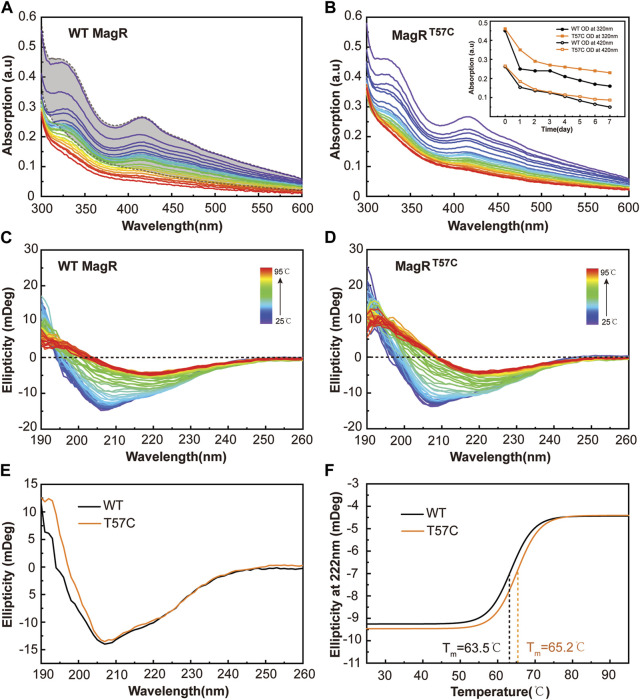
The clMagR^T57C^ shows prolonged iron-sulfur cluster stability. **(A–B)** UV–Vis absorption spectrum of wild type clMagR (WT, **(A)** and clMagR^T57C^ (T57C, **(B)** measured every 6 h at room temperature for continuous 7 days, showing time-dependent absorption decay of both proteins at different rate. The colors of the traces follow UV-Vis spectrum trend from purple (starting point) to red (ending point). The shaded area in **(A)** represents the range of UV–Vis absorption spectral of clMagR^T57C^ in 7 days (as in **B**) as a reference for comparison. The iron-sulfur cluster dissociation curves determined by absorption at 320 nm and 420 nm from **A** and **B** are shown as inserts in **(B) (C–D)** Thermal stability analysis of wild type clMagR (WT, **(C)** and clMagR^T57C^ (T57C, **(D)** measured by CD spectroscopy from 25 to 95°C. **(E)** Far-UV (190–260 nm) CD spectrum of wild type clMagR (WT, black line) and clMagR^T57C^ (T57C, orange line). **(F)** Thermal unfolding curves of wild type clMagR (WT, black line) and clMagR^T57C^ (T57C, orange line). Boltzmann sigmoidal fit of CD thermal denaturation curve at 222 nm depicting a melting temperature of 63.5°C for clMagR^WT^ and 65.2°C for clMagR^T57C^.

To further investigate if clMagR^T57C^ could stabilize the iron-sulfur cluster binding at ambient temperature, UV-Vis spectrums were measured with freshly purified clMagR^WT^ and clMagR^T57C^ for continuous 7 days parallelly at room temperature at the same concentration (200 μM), to monitor the iron-sulfur cluster bound in proteins. The loss of iron-sulfur cluster binding in protein was shown by the decrease in UV-Vis absorption. As shown in Figures 5A,B, the absorption peaks of clMagR^WT^ were much lower than that of clMagR^T57C^ after 7 days. It is obvious that wild type clMagR protein is losing its iron-sulfur clusters significantly faster than clMagR^T57C^. By mutating T57 to cysteine residue, the iron-sulfur cluster was stabilized in bound form in clMagR protein even at room temperature.

To further address the underlying mechanism of how T57C mutation leads to thermostability increase in clMagR, far-UV CD spectroscopy (190–260 nm) was applied to follow the unfolding and folding of proteins as a function of temperature from 25 to 95°C at 1°C intervals (Figures 5C,D) (Kanagarajan et al., 2021; Wensien et al., 2021). The clMagR^T57C^ mutant showed improved thermostability compared with clMagR^WT^ in the temperature range we recorded (Figures 5C,D). A side-by-side comparison of the secondary structure of both proteins at room temperature revealed largely unchanged profile between clMagR^WT^ and clMagR^T57C^ (Figure 5E), indicating that site-directed mutagenesis did not disrupt the overall structures of the protein, which is consistent with the size-exclusion chromatography result (Figure 3A). Further analysis showed that melting points (Tm) of clMagR^T57C^ was significantly higher than that of clMagR^WT^, suggesting the increased thermostability by mutating T57 to cysteine (Figure 5F).

## Discussion

Iron-sulfur proteins attracted much attention of protein design as they are of tremendous interest for their electron transfer properties and play essential roles in various fundamental biological processes (Nanda et al., 2016). The general purpose of re-designing an iron-sulfur protein could be to increase the stability in different environments and/or the sensitivity to redox changes, to explore its applications in protein-based therapies, or serve as biosensor, et al.

Most iron-sulfur proteins can be unstable when they are accessible to oxidizing substances (Rouault and Klausner, 1996). Some proteins such as pigeon MagR which contains relatively stable iron-sulfur clusters, mostly because that the cluster is bound in a region of the protein inaccessible to solvent and oxidants. Designing a more stable iron-sulfur binding site based on native protein has been very challenging (Nanda et al., 2016). One particular approach is to bury the cluster within the hydrophobic center of the protein by substitute nearby hydrophilic residues to hydrophobic residues, thus, to make the cluster inaccessible to oxidants. However, this approach could potentially damage their inherent susceptibility to redox changes. MagR, as a putative magnetoreceptor, its sensitivity to magnetic field changes could be modulated by different types of iron-sulfur clusters bound to the protein and regulated by redox cycle as well (Guo et al., 2021) . Therefore, we chose a different approach to stabilize the iron-sulfur cluster binding by adding an additional ligand to the cluster.

We screened various mutations in two hotspots around the iron-sulfur cluster binding site in pigeon MagR and found that most mutants we tested did not affect the type of iron-sulfur clusters bound to the protein. And most mutants also showed decreased iron-sulfur cluster binding efficiency and lower iron content compared with that of wild type protein. Only one particular mutation, T57C in clMagR showed improved iron-sulfur binding efficiency and iron content, without alter the type of bound iron-sulfur cluster. More importantly, prolonged thermostability has been found with clMagR^T57C^, and CD spectroscopy data also reveals an overall structural stability improvement.

The ultimate goal of protein engineering of MagR is to provide a molecular tool for magnetogenetics in the future with improved stability at ambient temperature and hypersensitivity to external magnetic field changes. Previously we rationally designed a single-chain tetramer MagR (SctMagR) as a building block to facilitate MagR assembly (Yang et al., 2022), and here in this study, we designed a better iron-sulfur binding site in clMagR^T57C^ with prolonged stability at room temperature. The work we presented in here, as well as in previous report, may serve as steady steps toward the magnetogenetics applications in the future.

## Method

### Expression and purification of clMagR^WT^ and its mutant proteins

The expression vector of the pigeon (*Columba livia*) MagR (clMagR) was constructed as previously described (Qin, Nature Materials, 2016). The expression vectors of the clMagR^E128A^, clMagR^E128C^, clMagR^G125_C126insK^, clMagR^T57C^, clMagR^R58C^ and clMagR^T57_R58insC^ were obtained by site-directed mutagenesis (TIANGEN) using clMagR^WT^ plasmid as template.

All proteins were recombinantly expressed in *E. coli* strain BL21 (DE3). Briefly, the protein expression was induced by 20 µM isopropyl β-D-1-thiogalactopyranoside (IPTG) overnight at 15°C (288K). Bacteria cells were harvested and resuspended in lysis buffer (20 mM Tris, 500 mM NaCl, PH 8.0) with complete protease inhibitor cocktail (Roche) and lysed by sonication on ice. The supernatant was collected after centrifugation and loaded onto a Strep-Tactin affinity column (IBA). The column was washed with washing buffer (20 mM Tris, 500 mM NaCl, pH 8.0) for approximately 30 column volumes (CV) to remove unbound proteins. Afer washing, proteins were eluted from Strep-Tactin affinity columns with elution buffer (20 mM Tris, 500 mM NaCl, 5 mM desthiobiotin, pH 8.0). PageRuler Prestained Protein Ladder (Thermo Scientific, Product# 26616) was used as the molecular weight standards for all SDS-PAGEs.

### UV-Vis analysis of clMagR^WT^ and mutant proteins

Wild type MagR and mutant proteins were prepared at 200 μM in TBS buffer (20 mM Tris, 150 mM NaCl, pH 8.0) and UV-Vis absorption measurements in the near UV visible wavelength (300–600 nm) were recorded using a nanodrop spectrophotometer (Thermo Scientific, NanoDrop OneC).

### Electron paramagnetic resonance spectroscopy

X-Band (∼9.6 GHz) EPR spectra were recorded on an EMX plus 10/12 spectrometer (Bruker, Billerica, MA), equipped with Oxford ESR910 Liquid Helium cryostat. For the oxidized protein samples, 200 μL of 1 mM as-isolated purified protein were mixed with 50 μl of glycerol in TBS buffer (20 mM Tris, 150 mM NaCl, pH 8.0). For reduced protein samples, 10 mM sodium dithionite (Na_2_S_2_O_4_) was added to the above protein solutions. Then, the protein samples were transferred into 4 mm diameter quartz EPR tubes (Wilmad 707-SQ-250 M) and frozen in liquid nitrogen. The EPR signals of oxidized and reduced proteins were recorded at different temperatures (10 K, 25 K, 45 K, and 60 K) with a modulation amplitude of 2 G, a microwave frequency of 9.40 GHz, an incident microwave power of 2 mW, and a sweep time of 25.60 s.

### Chemical reconstitution

A concentration of 400 μM purified (as-isolated) wild type clMagR (clMagR^WT^) or mutant proteins were incubated overnight at 4°C in TBS buffer (20 mM Tris, 150 mM NaCl, pH 8.0) with 10 mM EDTA and 10 mM sodium dithionite (Na_2_S_2_O_4_) to remove the bound iron-sulfur clusters. The mixture was desalted with a PD MiniTrap G-25 desalting column (GE Healthcare) and the obtained protein was labeled “apo clMagR”. Then, 5 mM DTT was added into the protein solution and incubated for 30 min at 4°C, followed by dropwise addition of fresh Fe(NH_4_)_2_(SO_4_)_2_ and Na_2_S both at 8-fold molar excess relative to protein. The mixture was incubated at 4°C overnight. The protein solution was then passed through a PD MiniTrap G-25 desalting column to remove unbound iron and sulfate, and the obtained protein sample labeled “chem re clMagR”.

### Circular dichroism spectroscopy

Circular dichroism (CD) was applied evaluate secondary structures in protein in the far UV range (190–260 nm) and to monitor protein-bound co-factors such as iron-sulfur clusters in the near UV-Visible range (300–600 nm) in this study. As for the protein-bound iron-sulfur cluster types analysis, purified wild-type MagR protein (clMagR^WT^) and mutants were prepared at 100 μM in TBS buffer (20 mM Tris, 150 mM NaCl, pH 8.0) and measured in 1 cm diameter quartz cells at room temperature using a MOS-500 (Biologic) CD Spectrometer. To analyze the secondary structure and thermal stability of wild type MagR and its mutants, measurements were performed using a J-1700 CD Spectrometer (JASCO Corporation, JPN) in the Far-UV range (190–260 nm). Purified wild-type MagR protein (clMagR^WT^) and mutants were prepared at 10 μM protein (20 mM Na_2_HPO_4_, pH 8.0) in 1 mm quartz cuvettes at room temperature. The thermal stability of wild-type MagR protein (clMagR^WT^) and mutants were monitored and recorded by CD spectrum in the range of 25°C to 95°C with temperature increases at 1°C intervals. The melting temperature values were calculated by sigmoidal fitting of the thermal denaturation curve at 222 nm using Boltzmann function.

### Ferrozine assay

Ferrous iron reacts with ferrozine (0.1% (w/v) ferrozine in 50% (w/v) ammonium acetate) to form an intense purple complex that can be quantified spectrophotometrically at 562 nm using a microplate reader. The iron (Fe) content in MagR and its mutant proteins was quantified by reducing Fe with hydroxylamine hydrochloride (10% (w/v) HAHCl in 1M HCl) and analyzed by ferrozine assay. Briefly, aliquots of protein and HAHCl mixture (80 μl HAHCl and 20 μl proteins at 100μM, total 100 μl) were incubated at 37°C for 30 min in the dark in a 96-well plate, then, 100 μl ferrozine was added into each well and incubated at 37°C for additional 15 min in the dark. The iron-ferrozine complex was measured at 562 nm on a microplate reader (Tecan Spark). A standard curve for ferrozine assay was generated using a series ferric chloride solution (0–500 μM) in 1 M HCl. The total iron content of MagR protein and its mutants were calculated by comparing its absorbance to that of a range of standard concentrations of equal volume that had been prepared in a way similar to that of the protein samples by linear regression analysis. There were three replicates for each protein sample. Histograms and statistical analyses were performed by using the software GraphPad Prism. Student’s t-test was used to test for differences in total iron between protein samples and considered significant at *p* <0.05.

## Data Availability

The original contributions presented in the study are included in the article/Supplementary Material, further inquiries can be directed to the corresponding authors.
