# Supplementary material for: The rational design of iron-sulfur cluster binding site for prolonged stability in magnetoreceptor MagR
Source: Front Mol Biosci. 2022 Nov 10;9:1051943. doi: 10.3389/fmolb.2022.1051943 (PMC9685556; doi:10.3389/fmolb.2022.1051943)
Supplement: Supplementary file 1 [file DataSheet1.PDF]

frontiers

Supplementary Material

Supplementary Figures

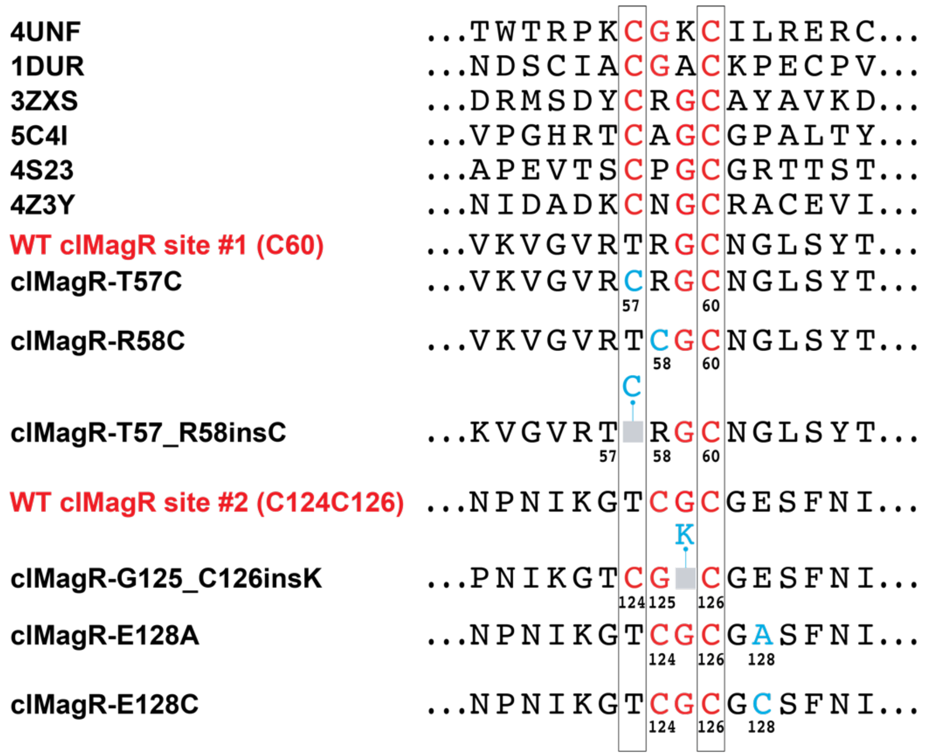

Supplementary Figure 1. Rational design of the iron-sulfur binding sites of clMagR. Sequences of iron-sulfur proteins (PDB IDs: 3ZXS, 4Z3Y, 5C4I, 4UNF, 4S23, 1DUR) contain Cys-X-Gly-Cys or Cys-Gly-X-Cys motifs in their iron-sulfur binding sites were aligned. Cys and Gly are highlighted in red. The iron-sulfur binding site of MagR can be divided into two parts and various site-directed mutagenesis have been designed and shown above. The mutated residues were highlighted in cyan.

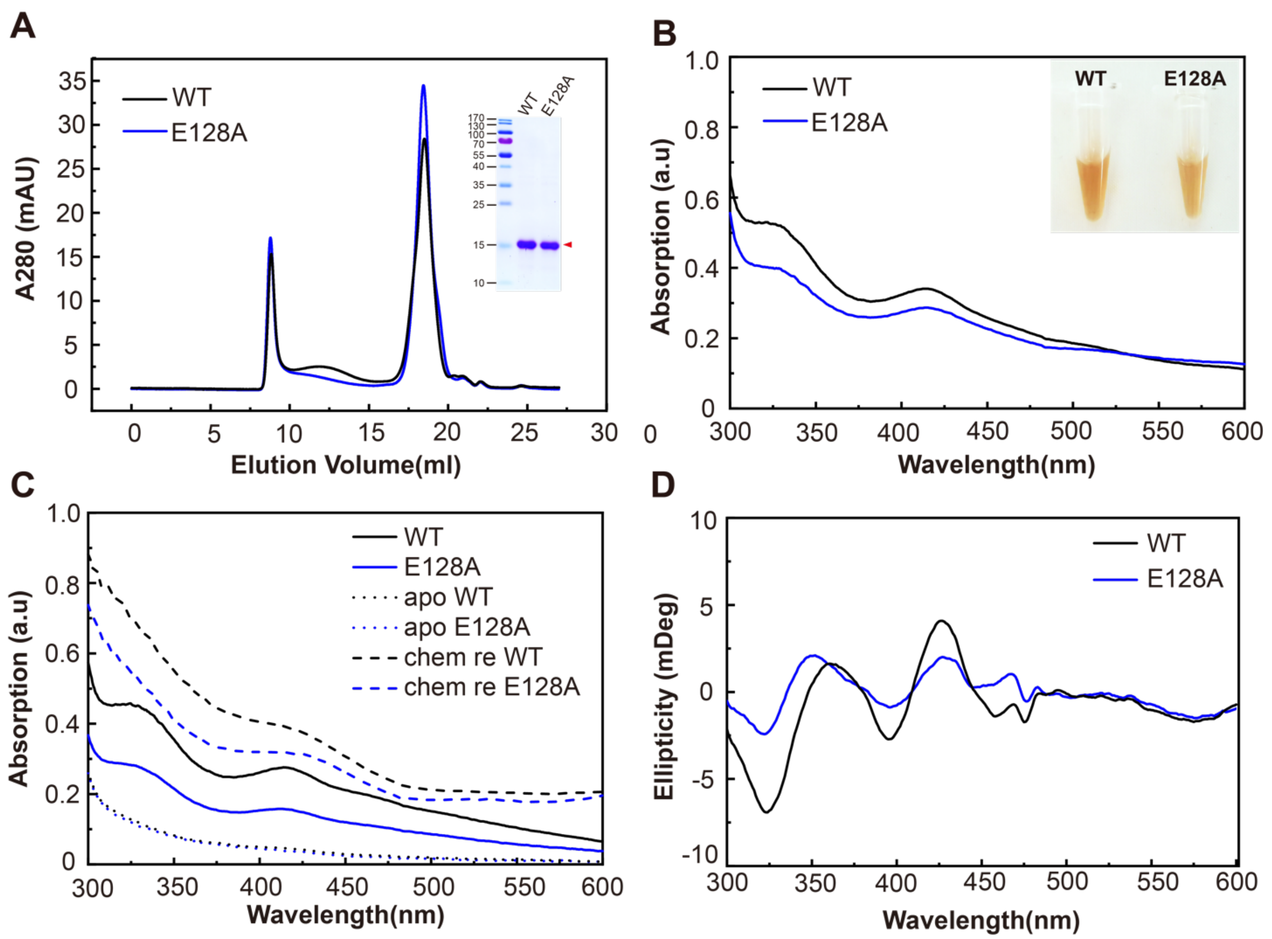

Supplementary Figure 2. The characterization of clMagR^{E128A}. (A) Size-exclusion chromatography of purified wild type clMagR (WT, black line) and clMagR^{E128A} (abbreviated as E128A, blue line). SDS-PAGEs of protein preparation are shown as inserts. **(B)** UV–Vis absorption spectrum of purified clMagR (WT) and its mutant (E128A), with the same color scheme as in a. **(C)** UV-Vis absorption spectrum of chemically reconstituted clMagR (WT) and its mutant (E128A). As-isolated proteins were shown as solid lines, apo proteins with iron-sulfur cluster removal were shown as dotted lines, and chemically reconstituted proteins (labeled as chem re) were shown as dashed lines. **(D)** Circular dichroism (CD) spectrum of wild type clMagR and its mutant (E128A).

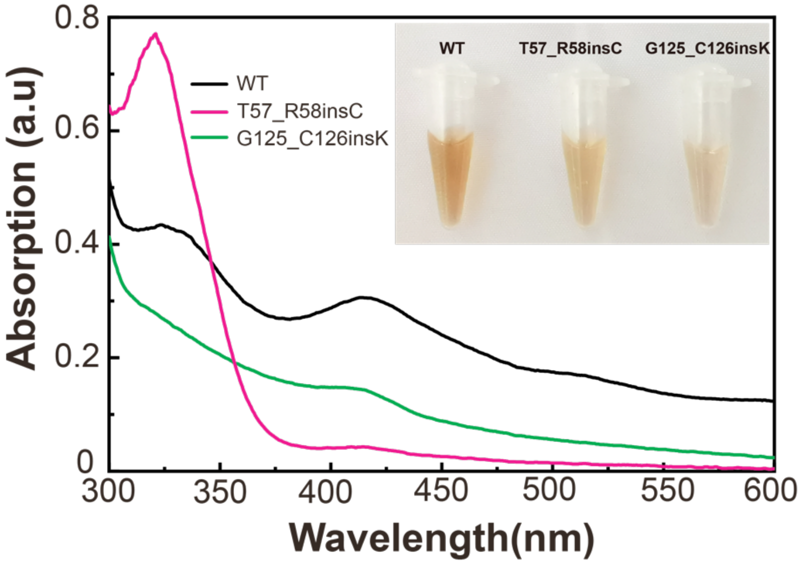

Supplementary Figure 3. UV-Vis absorption spectrum of wild type clMagR (black line) and two insertion mutants, clMagR^{T57_R58insC} (pink line) and clMagR^{G125_C126insK} (green line).

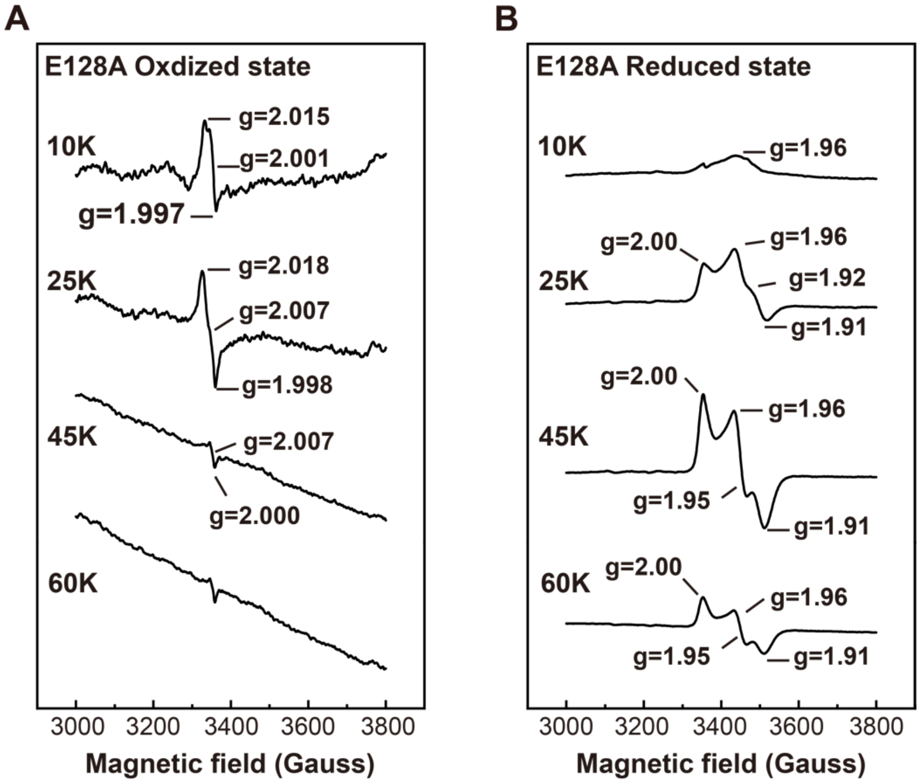

Supplementary Figure 4. EPR spectrum of clMagR^{E128A}. EPR were measured both at oxidized **(A)** status and reduced status **(B)**, and recorded at different temperatures (10K, 25K, 45K and 60K).
